# Complete Genome Sequence of Bacillus subtilis subsp. subtilis Strain 3NA

**DOI:** 10.1128/genomeA.00084-15

**Published:** 2015-03-12

**Authors:** Daniel R. Reuß, Jörg Schuldes, Rolf Daniel, Josef Altenbuchner

**Affiliations:** a Department of General Microbiology, Georg-August-University Göttingen, Göttingen, Germany; b Department of Genomic and Applied Microbiology & Göttingen Genomics Laboratory, Georg-August-University Göttingen, Göttingen, Germany; c Institute of Industrial Genetics, University of Stuttgart, Stuttgart, Germany

## Abstract

Bacillus subtilis 3NA reaches high cell densities during fed-batch fermentation and is an interesting target for further optimization as a production strain. Here, we announce the full genome of B. subtilis 3NA. The presence of specific Bacillus subtilis 168 and W23 genetic features suggests that 3NA is a hybrid of these strains.

## GENOME ANNOUNCEMENT

Bacillus subtilis 3NA, obtained from the Bacillus Genetic Stock Center (BGSCID 1S1), is extensively used in our laboratory for heterologous gene expression and high cell density fermentation (1, 2). The strain is a *spo0A* mutant isolated by Michel and Millet (3). Due to this mutation the strain produces no spores and low amounts of proteases. Despite the fact that the strain was characterized as nontransformable we found transformation frequencies comparable to B. subtilis 168.

To the best of our knowledge, 3NA is the only Bacillus strain which allows high cell densities during fed-batch fermentations (cell dry weight [CDW] up to 75 g/L). B. subtilis 168 and mutants, deficient in sporulation-specific sigma factors stopped growth already during the batch phase and reached CDWs between 5 and 15 g/L. For a better understanding of the phenotype and genetic optimization of 3NA as a production strain, the genome sequence was determined from 5.54 million reads obtained from an Illumina 2 × 75-bp paired-end run. The reads were aligned to the B. subtilis 168 genome (GenBank accession no. NC_000964 [4]) using the Geneious 6.0.3 Read Mapper included in the Geneious 7.1.7 software from Biomatters Ltd. (5). The final circular sequence has 4,195,102 nucleotides with 92× mean coverage and a 99% identity to the reference genome of B. subtilis 168. The genome of B. subtilis 3NA shows 425 variations (single nucleotide polymorphism [SNP], deletion, insertion, and substitution) with a minimal coverage of 25 and a minimum variant frequency of 0.8 compared to the B. subtilis 168 genome. A full list of all the variations can be obtained from the corresponding author on request.

The mutation of the *spo0A* gene could be confirmed. A frameshift mutation (−G) leads to an early stop codon. Further frame shift mutations were found in the *yvdK* and *putP* gene (maltose phosphorylase and proline permease). Another interesting mutation was found in the *abrB* gene. A base exchange in the translation stop codon elongates AbrB from 96 aa to 107 aa.

Two major differences between 3NA and B. subtilis 168 were observed. One is the lack of the integrative conjugative element ICE*Bs1* (6). The second one is a 6.4-kb region between *trpC* and *cheR* which contains 90.6% of all 425 single nucleotide polymorphisms identified between 3NA and B. subtilis 168. This region is highly homologous to B. subtilis subsp. *spizizen* W23.

According to Michel and Millet (3), 3NA is derived from the B. subtilis Marburg wild type. The presence of single base duplications in the genes *swrA*, *sfp* and a 9-bp duplication in *gudB* typical for B. subtilis 168 and a “W23 island” at the *trpC* locus indicate that 3NA is actually a B. subtilis 168-W23 hybrid as described before for other Bacillus strains by Zeigler et al. (7). The 6.4-kb size of the W23 island further indicates that the strain SMY described by Bohin et al. (8) might be the 3NA parental strain.

### Nucleotide sequence accession number.

The genomic sequence of B. subtilis subsp. subtilis 3NA is deposited in GenBank under the accession no. CP010314.
